# Stakeholders’ Roles in Evolutionizing Education: An Evolutionary-Based Toolkit Surrounding Elementary Education

**DOI:** 10.3390/bs15010092

**Published:** 2025-01-20

**Authors:** Kathryne Gruskin, Mariah Griffin, Sonaskshi Bansal, Stephanie Dickinson-Frevola, Ashlee Dykeman, Desiree Groce-Volinski, Keydy Henriquez, Maya Kardas, Aileen McCarthy, Aman Shetty, Brandon Staccio, Glenn Geher, Ethan Eisenberg

**Affiliations:** Department of Psychology, State University of New York at New Paltz, New Paltz, NY 12561, USA; griffinm6@newpaltz.edu (M.G.); bansals1@newpaltz.edu (S.B.); dickinss2@newpaltz.edu (S.D.-F.); dykemana2@newpaltz.edu (A.D.); grocevod1@newpaltz.edu (D.G.-V.); henriquk7@newpaltz.edu (K.H.); kardasm1@newpaltz.edu (M.K.); mccartha8@newpaltz.edu (A.M.); shettya4@newpaltz.edu (A.S.); stacciob1@newpaltz.edu (B.S.); geherg@newpaltz.edu (G.G.); eisenbee1@newpaltz.edu (E.E.)

**Keywords:** evolution, education, psychology, school reform, evolutionary mismatch

## Abstract

There is a rapidly growing body of research in the field of evolutionary educational psychology that examines children’s evolved motivational and educational inclinations as they relate to modern learning and schooling. It is generally agreed that schools are inherently mismatched with how children of our species evolved to learn, thereby contributing to difficulty learning and associated adverse schooling outcomes. Many researchers argue that, by making small changes to schools that help to better align instructional methods and childhood as a whole with our species’ evolved learning mechanisms, we can lessen the negative impacts from evolutionary mismatch and create better outcomes for modern students. In order to create effective change, there must be collaborative work done by parents, teachers, and school administrators. This paper delineates the roles of these stakeholders in elementary education with respect to creating more evolutionarily relevant systems. A research-based toolkit is proposed to guide these stakeholders in evolutionizing the elementary education system.

## 1. Introduction

Modern 21st century schools are responsible for providing the skills and knowledge that children will need to be successful adults. Elementary schools, serving children ages four to eleven, are tasked with laying the foundation for this knowledge and all the learning that students will do over the course of their educational careers. As such, educational institutions play a central and critical role in nearly all westernized childhoods. In the United States alone, roughly 49.6 million children attended public school for kindergarten through twelfth grade in 2022 (NCES, 2024). Understanding the importance of education and the vast number of students served, it follows that there is significant and ongoing debate regarding the best manner in which to educate all students. A high-quality education is essential to equip children with the skills necessary for success in their adult lives.

That said, there is no consensus on the best way to educate all elementary aged children in the United States. Rather, the modern schooling landscape varies greatly and is rife with ongoing debate and disagreement (Barrs & Rustin, 2017; McGrath, 2017). While most children in the United States attend public schools, there are also large percentages of children who attend private schools or other alternative school settings (NCES, 2024). There are large, often politically fueled, debates regarding curricula, standards, school funding, etc. that vary from state to state and even from district to district (Barrs & Rustin, 2017). Much of the decision-making regarding education often comes from non-educator policymakers (McGrath, 2017). Education reform, as a whole, is difficult to achieve (Farrell, 2000). In sum, modern American schools lack uniform guiding principles that ensure equity and quality education for all children, regardless of location or school.

While many fields have weighed in on educational reforms (see Barrs & Rustin, 2017; Farrell, 2000), one growing voice comes from the field of evolutionary educational psychology. As a sub-branch of evolutionary psychology, evolutionary educational psychology posits that, by understanding the evolutionary history of our species as it relates to learning, we are better able to adapt modern instruction to meet our evolved learning needs and preferences (Geary, 2002). Evolutionary educational psychology can be thought of in terms of Niko Tinbergen’s (1963) ideas of ultimate and proximate explanations of behavior. The ultimate underpinnings of learning refer to the ways in which our species, as hunter-gatherers, first evolved to learn through play, collaboration, and exploration. The proximate drivers of learning reference the ways in which those evolved mechanisms play out in modern learning environments. In other words, the unique contribution of evolutionary educational psychology is the power to understand children’s evolved learning mechanisms as they relate to modern instructional practices. Using evolutionary educational psychology to inform pedagogy has the potential to create better outcomes for modern students by helping to promote success, both throughout and after the schooling experience.

The following sections of this paper will review much of the existing research in the field of evolutionary educational psychology. As this field is rapidly growing (see Geary & Xu, 2022), there is a need to begin to synthesize this work into meaningful takeaways that can be used by stakeholders within the education system to create evolutionarily informed change. In the context of this work, the term stakeholder is used to refer to parties that have a vested intertest in the educational system. Specifically, the stakeholder groups discussed in this paper are parents, teachers, and administrators. This paper aims to propose toolkits—i.e., practical recommendations with interactive prompts—that can be used by these stakeholders to better align modern elementary educational experiences with evolved learning mechanisms. Importantly, the toolkit helps to guide the three groups of stakeholders to work both independently and collaboratively to create the best possible outcomes for students. Parents are guided to reflect on their parenting styles and the ways in which they support their child’s school experiences. Teachers are tasked with looking at their instructional choices and teaching philosophies. Lastly, administrators can reflect on their roles in supporting the school environment and classroom instruction. To make impactful change, all stakeholders must be invested in evolutionizing education—that is, making education more evolutionarily aligned.

While the general purpose of this paper is to add to the literature on improving elementary education in a broad sense, the specific point is to address how an evolutionarily informed approach may have the capacity to shed unique and powerful light on issues related to elementary education. An understanding of evolutionary educational psychology has the potential to aid parents, teachers, and administrators in better understanding how children have evolved to learn best and how those evolved learning mechanisms can be used to inform pedagogical decision making.

## 2. Basic Features of Ancestral Education

To understand how evolutionary educational psychology can be used to help inform modern instructional decision making, it is first important to understand the conditions under which our species evolved. This understanding of how evolution has shaped learning helps to guide the theory behind the toolkit prompts and suggested educational practices.

Evolutionary psychologists refer to the conditions under which a species primarily evolved as the environment of evolutionary adaptedness, or the EEA for short (Bowlby, 1969). The EEA conditions include the selective pressures as well as the survival or reproductive hurdles that a species would have faced over evolutionary time. Due to the extended time and the significance of these pressures in the EEA, natural selection likely shaped bodies, minds, and behaviors to contend with these conditions (see Geher, 2014).

For the lion’s share of evolutionary history, humans and our hominin ancestors lived in nomadic hunter-gatherer tribes (see Geher, 2014). These tribes were generally small—less than 150 people—and the members of the tribe were all closely related to one another (Dunbar, 1992). Members of these tribes survived on a subsistence lifestyle, with much of their time and energy being devoted to tasks such as child rearing, hunting, and gathering food. It was not until roughly 10,000 years ago that cultural evolution begin to advance rapidly and our ancestors transitioned to a more sedentary, agricultural-based lifestyle.

Much of what we know about EEA conditions comes from studying extant hunter-gatherer tribes (e.g., the Hadza of Tanzania; see Hawkes et al., 1991). Researchers—unable to go back to see the conditions in the EEA—have used these tribes as a proxy of what life was like for our human ancestors. While each tribe studied is unique, there are underlying similarities that have made these tribes a generally reliable source of knowledge about likely conditions for human ancestors (Konner, 2007).

Studying children in these cultures has led to an understanding of ancestral education (Gray, 2009; Lancy et al., 2010). The primary takeaway from this work is that none of the cross-cultural, hunter-gatherer studies reported any formal system of education that remotely resembles western schools (Gray, 2009, 2013). Instead, education and learning were natural, ongoing activities for children and adults alike.

While all tribes showed extensive evidence of education and learning, the concept of schooling was nonexistent. Taken at its most basic definition, learning is anything that creates a lasting change in behavior or thought (Bjorklund, 2021). Conversely, schooling is the formal process of explicitly educating children. Schooling and explicit teaching is only found in WEIRD (westernized, educated, industrialized rich, democratic) societies (Lancy, 2015). Humans, due to our highly social nature and complex culture, need to learn significant amounts of information to achieve success as adults. As a result of the importance of social relations and learning in the EEA, our brains have been shaped by natural selection to acquire knowledge in a manner related to how our ancestors first evolved to learn (Bjorklund, 2021).

Drawing on the work of Peter Gray (2009), we have a strong understanding of what learning likely looked like for our ancestors. Nearly all learning in the EEA would have been child-led. As the adults were busy with work related to finding nutrition and other daily living tasks, the children were left essentially to their own devices. From the time children were weaned from their mother’s breast milk at about four years of age to the time they fully contributed to the tribe as an adult, at approximately 17 years of age, children would engage in mixed-age, collaborative play. Learning was accomplished by observing the actions of the adults and recreating or mimicking those actions through play. There were no child-specific toys in the EEA. Children would borrow tools from adults and learn to appropriately use these items through play and collaboration. Rather than having dedicated adult teachers, children would support one another as they developed the skills they saw valued in their local tribe. Lastly, children in the EEA developed at their own pace. There were no standards or assessments driving instruction. Instead, education was driven by an innate desire and motivation to learn essential skills (Gray, 2009).

Further evidence supporting evolved mechanisms for learning can be found in the neurobiological research of play-based learning. Panksepp (1998) proposes that play is one of the primary process systems in the brain that underlies behavior and emotions in mammals. Research into the neural circuitry of play supports the idea that play evolved early on in evolutionary history (Panksepp, 1998). Play arises from ancient regions of the brain to motivate and aid in the programming of higher brain regions that relate to social strategies and learning (Kellman & Radwan, 2022; Panksepp, 2010).

In sum, human childhood, characterized by a prolonged period of immaturity and childhood propensities towards play and social learning, was shaped by natural selection to facilitate the acquisition of critical skills and knowledge (Bjorklund, 2021).

## 3. Primary and Secondary Knowledge

To reiterate, the purpose of modern schools is to provide children with the skills they will need to be successful adults. Learning under ancestral conditions was achieved with the same purpose; however, necessary skills were more focused on overcoming immediate survival and reproductive hurdles. To make any evolutionarily-based reforms to the modern education system, it is first important to understand the different types of knowledge and skills from an evolutionary perspective and how these skills are utilized in the context of one’s culture and environment.

Evolutionary psychologist David Geary (1995) proposes that humans have evolved mechanisms to readily obtain knowledge and skills centered upon the goals of survival and reproduction. Geary calls this type of information primary knowledge. Primary knowledge, or primary skills, can be learned through unconscious observation and play, rather than through explicit instruction. For example, knowing how to speak and understand one’s own native language is an example of primary knowledge, as this skill is important for success and survival. Learning to speak is observational and intuitive; it is something babies learn without being taught. Geary further classifies primary knowledge into folk domains related to biology, psychology, and physics. Folk biology relates to navigating challenges related to the natural world. Folk psychology centers upon an individual’s sense of self and interpreting relationships with others. Folk physics deals with problem-solving and a general understanding of navigation and material objects (Geary, 2008).

Conversely, modern schooling places more emphasis on acquisition of secondary knowledge than primary knowledge (Geary, 2012). Secondary knowledge refers to information and skills that can only be obtained through explicit instruction, such as higher order math skills or learning to read and write. Secondary knowledge developed much later in the evolutionary timeline of our species and these skills are more related to cultural transmission than to survival and reproduction (Geary, 2008). The ability to acquire these skills has likely not been shaped by natural selection. This idea is further supported by John Sweller’s *Cognitive Load Theory* (Sweller, 2011): Secondary knowledge is slower to develop than primary knowledge. This slower knowledge acquisition requires conscious effort and the use of working memory, as the information being taught is often not intuitive or instinctual.

The shift in emphasis towards secondary knowledge and skills corresponds with modern society’s focus on preparation for career and financial success over basic survival and reproductive success. However, that emphasis on secondary knowledge leads to challenges within the education system. In one study conducted by Lespiau and Tricot (2022), researchers examined the different effects of instruction in primary versus secondary knowledge on motivation and working memory. It was found that a focus on primary knowledge supports academic motivation and performance when faced with various cognitive load tasks. However, it is possible to leverage primary knowledge and skills to more effectively teach secondary skills. It was reported that the presentation of primary knowledge before secondary knowledge had a positive impact on learning by both increasing the learners’ confidence in their answer and decreasing the perceived cognitive load (Lespiau & Tricot, 2022). Additionally, access to primary knowledge can be used to increase learners’ motivation to learn secondary skills. For example, learning to read is a secondary skill. On its own, learning to read is not likely to be an enjoyable or highly motivating activity for children. However, children are motivated to learn to read because reading grants access to content that reflects primary information (i.e., social relationships and competition) (Geary, 1995).

An understanding of both primary and secondary knowledge is important for developing best practices in education. Considering the skills and knowledge that can be learned through observation and social learning prior to—or in the absence of—explicit adult instruction and those than cannot is critical to implementing evolutionarily informed change. Stakeholders can use these ideas of primary and secondary knowledge to inform the amount of adult involvement needed in childhood learning, and this is reflected in the toolkit prompts.

## 4. Evolutionary Mismatch

In modern classrooms, learning looks very different from the learning of hunter-gatherer tribes (Gray, 2013). Western children lack exposure to aspects of their evolved learning inclinations, such as age-mixing, play, and self-directed learning. With our modern public education system, children are forced to learn and play—if there is time for play—in classrooms with children of the same age. They are given a directed curriculum. Most of their day is spent inside, doing sedentary desk work. On one hand, children today do need more explicit instruction to be able to master necessary secondary knowledge and skills. On the other hand, our brains still expect to learn primary skills in conditions similar to those of the EEA. This difference between current conditions and conditions we have evolved to expect is referred to as evolutionary mismatch (see van Vugt et al., 2020).

Evolutionary mismatch can be seen across various aspects of modern life. Diet and exercise are two ways in which we experience evolutionary mismatch. In each case, there are adverse effects from high levels of mismatch. For a highly processed diet, unlike what was experienced in the sustenance lifestyles of our ancestors, there are negative health outcomes, such as obesity, heart disease, and other complications. From sedentary lifestyles, we experience similar negative effects. Taking steps to decrease the evolutionary mismatch helps improve outcomes—e.g., reducing our intake of processed foods and adding more movement to one’s day (Geher, 2014).

Many of the issues that we see in education are likely at least partially influenced by the levels of evolutionary mismatch. Children are unhappy, there are increasing levels of childhood mental health issues, they lack key social–emotional skills, and academic outcomes are poor (see Bjorklund, 2021; Gray, 2013; Gruskin & Geher, 2018); at least in part, these negative outcomes may be driven by evolutionary mismatch, as well as an over emphasis on acquiring secondary knowledge and an underemphasis on primary skills (as described previously). The toolkit prompts help stakeholders to both be aware of and take steps to decrease instances of evolutionary mismatch in education. Doing this will likely help to address some of the above issues within the modern education system.

## 5. Evidence of the Effectiveness of the Evolutionary Approach to Education

There is a growing body of evidence from the field of evolutionary educational psychology that a more evolutionarily-relevant childhood education improves outcomes for modern children (see Bjorklund, 2021). An evolutionary approach to childhood education keeps in mind the evolved learning needs of students, primary and secondary knowledge and skills, and evolutionary mismatch in modern school settings. All of these ideas are used to make instructional decisions that best meet the needs of students through an evolutionary lens. Such instructional decisions include an emphasis on student collaboration, active or hands-on learning, the student voice in learning, real-world connections or learning, and an emphasis on play. There are several types of alternative school models, highlighted below, that make use of these evolutionarily informed ideas. There are also many examples of alternative pedagogies that, despite never becoming standard in classrooms, work to reduce some instances of evolutionary mismatch. As a result, we end this section with our proposal for a toolkit outlining what we believe to be impactful and feasibly implemented evolutionarily informed pedagogy.

Some researchers (e.g., Gray, 2017) advocate for a completely reformed education system, such as that seen in the Sudbury Valley School in Framingham, Massachusetts. In this alternative school, children learn completely from freely age-mixing and engaging in self-directed activities. The school is an open campus, with materials for learning and teachers who support students only as needed or requested by students. There is no explicit formal curriculum. Rather, students at the Sudbury School primarily learn through collaborative play and exploration (Gray, 2011).

A more common example of an alternative school model is found in Montessori schools. Montessori Schools and their underlying philosophy mix aspects of contemporary formal education and aspects of nomadic learning. Children engage in self-directed and hands-on learning, with natural tools and adult guides. According to Lillard (2013), in Montessori schools, teachers typically explain a given topic and its underlying theory while the children follow along with a related hands-on activity. This is considered the structured, more formal part of this type of education. The teacher then provides a plethora of activities that fall under the given topic, and the children freely choose the activity in which they would like to engage. This exploration can be considered the more unstructured, evolutionarily-relevant part of the Montessori model. By mixing structured and unstructured pedagogy, students are better able to acquire both primary and secondary skills than if they were exposed to just one form of pedagogy.

There have been wonderful documented outcomes for Sudbury Schools (Gray, 2013) and Montessori schools (Lillard et al., 2017; Lillard, 2018). Children who attended these schools achieved similar, if not better, academic achievements as their peers in traditional school settings and have been documented to have successful college experiences and careers post-schooling (see Gray, 2013; Lillard et al., 2017; Lillard, 2018). However, it is unlikely that we will see such sweeping reforms in public education any time soon. Instead, it is possible that small changes can shift the needle toward more evolutionarily-relevant learning methods within the constraints of the public school system. Doing so would feasibly lessen the quantity of evolutionary mismatch in schools and thereby lead to better outcomes for students.

There have been innumerable alternative pedagogies proposed and utilized in public school classrooms throughout history—such pedagogies include multi-age classrooms (Carter, 2005; Cohen, 1990), inclusive education (Schnepel et al., 2024), co-teaching (Barron & Friend, 2024), and personalized learning through technology use (Van Schoors et al., 2021). However none of these pedagogies, despite decreasing some instances of evolutionary mismatch in schools, have become standard practice in modern classrooms. Partly, this is due to the difficulty in enacting sweeping educational reforms in current contexts (Farrell, 2000). However, resistance may also be due to the disjointed nature of these ideas. While each pedagogy above has empirical support, they lack a uniform theory to guide implementation. This is where evolutionary educational psychology has the power to inform multiple areas of pedagogy and connect proximate learning behaviors to their ultimate evolutionary roots (see Tinbergen, 1963).

One study that examined the potential effects of implementing evolutionarily informed pedagogy was conducted by evolutionary psychological researchers Gruskin and Geher (2018). In a survey of college students, it was found that students who recalled more evolutionarily-relevant teaching and learning methods in their early education reported higher levels of success throughout their educational careers. This success included greater school enjoyment and higher grades from early childhood into their current college experiences. Importantly, nothing proposed by Gruskin and Geher is too detached from the norms of typical elementary education. Instead, the authors emphasized existing and feasibly implementable strategies that teachers could almost immediately use in their classrooms, in order to help decrease adverse effects from evolutionary mismatch.

The current paper complements Gruskin and Geher’s (2018) study by expanding on the ideas of an evolutionarily relevant elementary education and outlines the roles of stakeholders (parents, teachers, and administrators) in making evolutionarily informed changes to the educational system. As demonstrated in the evidence presented above, evolutionary educational psychology has the power to inform instructional practices in modern schools by capitalizing on children’s evolved educational mechanisms. There is much research that illustrates what this may mean in practice, but the evolutionarily based strategies and toolkit (Appendix A) proposed in this paper act as a clear guide for stakeholders to make meaningful and realistically implemented evolutionarily informed decisions about parenting and schooling. The following sections contain the literature and thought that guided the creation of the toolkit. The toolkit supports stakeholders in reflecting on their practice. The two components are intended to work side-by-side as a guide for stakeholders to evolutionize education.

Existing research shows that when all educational stakeholders are working towards the same goals, outcomes for students tend to be better than for any one party working in isolation (Rubinstein & McCarthy, 2010; Winthrop et al., 2021). By outlining strategies and creating an evolutionarily-relevant toolkit, we aim to outline the steps that must be taken by all stakeholders to meet the common goal of making elementary education more evolutionarily relevant, and thus, more effective in preparing children for success throughout their lives.

## 6. Education: Step One for All Stakeholders

The first step for all stakeholders, who are invested in making evolutionarily-relevant changes, in a child’s education, must be to educate themselves on the interconnectedness of evolution and education. By understanding the contexts in which children evolved to learn and how these evolved mechanisms impact modern education systems, parents, teachers, and administrators will be better equipped to create an evolutionarily informed schooling experience. However, many of the stakeholders are likely unaware of the evolution/education connections outlined in the present theoretical paper. As a result, education stands as one of the major barriers to creating a more evolutionarily relevant education system.

It is difficult to receive an education on evolutionary concepts. In one study by Glass et al. (2012), researchers across a variety of disciplines reported that it is very difficult to receive an education in evolutionary-related concepts at their higher education institutions. In addition, participants reported that most of their own evolution education was self-directed. This lack of exposure holds true in the field of education (Bjorklund, 2022). To effectively understand and utilize the ideas of evolutionary educational psychology, there is a need to address these ideas and strategies in higher education institutions and teacher preparation programs. The toolkit can act as a guide to doing so.

It is important to note that good pedagogy can happen without an explicit understanding of evolutionary concepts. Yet, an understanding of children’s evolved learning mechanisms helps stakeholders prioritize evolutionarily-aligned child-rearing concepts, instructional strategies, and policy decisions. As explained by David Bjorklund, “having an underlying theory that informs teachers and parents about how children evolved to learn will result in more enlightened teaching methods that will result in more enjoyable and successful learning experiences for children” (Bjorklund, 2022, p. 2266). The toolkit from this paper, as well as the broader ideas from evolutionary educational psychology, provide exactly that underlying theory to create better experiences for modern children. Thus, an education in evolutionary concepts is an essential first step for all stakeholders.

## 7. How Parents Can Evolutionize the Learning Process

Parents and guardians play a unique role in evolutionizing education. On one hand, parents are not directly responsible for the vast majority of school-based educational decisions and are typically not working in a school setting day-to-day. On the other hand, parents are responsible for the upbringing of children as well as the home environment, both of which play critical roles in the educational development of children. In addition, it is important to consider that education is not a school-only process (Gray, 2013). Learning, especially in childhood, is a natural and ongoing process that parents can support at home. With an evolutionarily informed awareness of child development, parents and caregivers can support the work being done in schools to reduce the adverse effects of evolutionary mismatch and develop a more evolutionarily informed parenting style.

### 7.1. Upbringing and Home Support

On the part of parents, evolutionizing education begins early, before children are even of school age. Early child rearing and the home environment set the stage for later schooling and learning. To create a positive and evolutionarily informed upbringing for children, parents should be aware of the habits and attitudes that they model, the use of technology in the home, and the ways in which they can be involved in and support their child’s education.

Due to the early importance of learning through observation, children have evolved a sensitivity towards the behaviors and attitudes of important adults in their lives (Bjorklund, 2021). Therefore, parents can offer emotional and cognitive support for children by modeling behaviors and creating safe, nurturing home environments. These environments help children develop strategies to cope with stress and emotional challenges that can arise later within the school setting (Bjorklund & Ellis, 2014). Parents can also support the development of their children by modeling good habits and attitudes. One study found that parental anxiety can have negative implications for school-aged children (Burstein & Ginsburg, 2010). Parental behaviors are essentially transmitted to their children, and outward anxiety from a parent can create childhood anxiety. This anxiety can impact the child’s cognition. Such anxiety was found to not only negatively impact children’s wellbeing, but also academic areas such as exam performance (Horn & Dollinger, 1989).

Monitoring technology is another vital component of evolutionizing education. The current reliance on and comfort with technology use, even at early ages, represents an instance of evolutionary mismatch that has critical implications for a child’s development (see Haidt, 2024). Parents should thus be aware of both the quantity of screen time and children’s online activities. Children evolved to learn through action and hands-on experiences, but now spend significant time using hand-held devices (e.g., smart phones, tablets). In the United States, it is estimated that children under twelve spend, on average, four to six hours per day watching or using screens. That number jumps to nine hours for teens (AACAP, 2024). It is important to note that this is a very novel cultural shift. Therefore, the effects on early childhood development may take many years to fully understand. Nevertheless, many researchers have focused on illuminating the negative mental health trends in children that are likely related to technology use (Haidt, 2024; Twenge & Campbell, 2019).

Additionally, when children are working exclusively with technology, they are working against their evolved instincts for activity and hands-on and social experiences (Bjorklund, 2021). In fact, children may not be learning as much from screens as many believe. There are several studies highlighting that children do not learn as well from 2D representations (e.g., videos) as they do from real-world, 3D, experiences (Barr, 2010, 2013). This is considered a video deficit: Learning outcomes from 2D representations are worse than learning outcomes from real-world objects or people (see Bjorklund, 2021). Further, technology use and screen time has been documented to lower children’s executive functioning skills (Lillard et al., 2015), which are necessary for planning and organizing, and has been associated with decreased white matter (myelin) in certain areas of the brain (Hutton et al., 2019)—that is, decreased efficiency of neural functioning. Technological boundaries in the home are critical for developing positive mental health and supporting learning for children. Parents should keep this evolutionary mismatch in mind and create technological limits and screen-free activities for children. The American Academy of Pediatrics recommends no technology use for children under two, up to one hour of screen time for children ages two through five, and continued boundaries (albeit more flexible ones) for older children (American Academy of Pediatrics, 2016).

Lastly, parental investment in education is essential. Parents can help reduce evolutionary mismatch by being actively involved in their children’s academic lives and working with other stakeholders to ensure that educational experiences align with their children’s developmental stages and evolved learning mechanisms. Moreover, parents can serve as advocates for educational policies that prioritize flexible, student-centered approaches to learning that better support children’s evolved learning needs (Steinberg, 2001). That said, it is important for parents to prioritize their children’s holistic development, not just their academic development. Parents can play a vital role in fostering skills by creating opportunities for children to pursue self-directed learning and play outside of school (Gray, 2011).

An evolutionarily informed parenting style focuses on modeling positive attitudes and behaviors at home, monitoring and limiting evolutionary mismatch from technology usage, and supports parent involvement in the learning and schooling process.

### 7.2. Childhood Empowerment

In addition to the above, it is important to recognize that a core purpose of child rearing and parenting is to aid in facilitating not only children’s cognitive development, but the development of an empowered, adaptive personality and agency over one’s life (see Bjorklund, 2021; Geary & Flynn, 2001; Gray, 2013). Throughout evolutionary history, parenting served as a mechanism to increase the likelihood of survival for human children who had a prolonged period of immaturity (Geary & Flynn, 2001). As discussed above and highlighted in the work of Peter Gray (2013), adults in hunter-gatherer tribes would have devoted most of their time to survival-related tasks, rather than direct supervision of children. Children were trusted to learn and explore with other children—without the direct supervision of adults. Parents did not directly dictate all activities of a child’s day, but rather provided flexible guidance as necessary (Gray, 2013). This is starkly contrasted with the direct role that parents play in their children’s lives in modern westernized societies.

In very recent history, there has been a massive shift in the trust and autonomy given to children (see Haidt, 2024; Twenge, 2017). Haidt refers to this shift as a rise in safetyism: an excessive concern for both the physical and emotional safety of children (Lukianoff & Haidt, 2018). This concern really began in the 1980s and has led to decreased freedom and decision-making autonomy for children. Rather than having the option to simply go and play in the local neighborhood after school, childhood today is ruled by structured and supervised play dates, clubs, and other adult-guided activities.

On one hand, today’s westernized world does pose many nuanced dangers and risks for children (Bjorklund, 2021). There is an unarguable need to protect children and provide reasonable limits on their freedoms. On the other hand, there are also important outcomes of such safetyism that should be considered. In a study of adolescent behaviors (Twenge & Park, 2019), it was reported that adolescents in recent years have shown decreased engagement in typical adult-like tasks, such as obtaining a driver’s license, trying alcohol, dating, or working for pay during the school year. While it may seem beneficial that children are being cautious and acting as children for longer periods, it also raises concerns over the development of agency—taking an active rather than passive role in one’s own life—during adolescence (Sercombe, 2014).

Children cannot be expected to make all decisions independently. That said, it is argued that children should be able to make their own choices, within reason, to help develop agency (see Gray, 2013). Examples could include the ability to play outside with peers in the neighborhood or, if not possible, in backyards or homes with adults nearby but not constantly monitoring play. Children should be able to make decisions about how and when (not if) to complete homework or school-related tasks. Further, children should be able to decide their interests (e.g., arts, music, sports) and pursue those interests in their own self-directed manner. Rather than dictating minute-to-minute schedules for children, parents can support the development of agency by allowing children some forms of reasonable freedoms. Gray (2013) refers to this type of parenting as trustful parenting.

Additionally, childhood is a key time for the development of self-efficacy: beliefs about one’s capabilities and competence (see Riggio et al., 2010). Higher self-efficacy has been shown to generalize to increased motivation and performance in tasks during childhood and into adulthood (Bandura, 1997). Household responsibilities during childhood (e.g., running errands, helping with cooking, and engaging in daily chores) have been shown to positively impact both general self-efficacy and work self-efficacy in young adults (Riggio et al., 2010). In the EEA, children would likely have begun contributing to daily living tasks of their hunter-gatherer tribes as they became proficient (Gray, 2013). Allowing children in modern societies the same opportunities to develop skills and contribute to their households will help develop critical and lasting self-efficacy beliefs.

An evolutionarily informed parenting style focuses supporting on a child’s development of agency and self-efficacy through empowerment and encouragement of reasonable risk taking. Encouraging these behaviors helps reduce adverse effects from the evolutionary mismatch of excessive safetyism.

### 7.3. Unstructured Activities and Play

Lastly, evolutionarily informed parenting focuses on children’s development through self-direction and play. Both structured and unstructured activities can aid in developing children’s executive function. Executive functions, such as organizing and inhibiting behavior, as well as employing working memory and cognitive flexibility, are cognitive skills that aid in self-control and meeting goals, and they develop primarily during childhood (Barker et al., 2014; Meltzer, 2018). Structured after-school activities may help develop executive functions (e.g., organization and inhibition): Children need to arrive on time, often have materials or practice prepared in advance, and inhibit urges to do something else that may be more immediately rewarding because they have previously committed to the after-school activity, likely working toward some long-term goal that requires short-term discipline (e.g., a school play or a sporting event). A child’s executive function can predict positive life outcomes, such as physical/psychological well-being and career success, both of which clearly require organization and inhibition (Barker et al., 2014; Meltzer, 2018). Organization and inhibition, in this case, are often driven by demands from parents and teachers. They are externally driven (Barker et al., 2014).

However, executive functions may also develop from internally motivated goals arising from unstructured after-school activities (Barker et al., 2014). After having asked parents to report the activities of their six- and seven-year-olds’ daily, annual, and typical schedules, Barker et al. (2014) found that children who spent more time in unstructured activities had better self-directed executive function than children who spent more time in structured activities. Visiting family, group activities such as walks or bicycle rides with friends, camping, picnicking, and free reading were all coded by Barker et al. (2014) as unstructured activities. In a verbal fluency task, children who were reported spending more time in those unstructured activities were better able to generate words of a certain category and efficiently decide on their own to switch from one subcategory to another. That is, the cognitive flexibility demanded by the task was stronger in children who engaged in more self-directed activities.

Unstructured play is also a critical learning experience for children and opportunities to engage in such play should be supported by parents. By engaging with peers, without the direction of adults, children practice and learn critical skills such as autonomy, sharing, and consensual decision making (Gray, 2009). Gray gives the example of a neighborhood baseball game rather than a Little League game. Voluntary participation of the players has significant impacts on the game and the required skills. Children in a pickup game have the option to freely play or leave, thereby getting along and playing cooperatively becomes critical for the continuation of the game. Gray writes, “if players were compelled to stay in the game, then the more powerful players could dominate, and the autonomy, equality, sharing, and consensual decision making would be lost” (Gray, 2009, p. 486). By allowing opportunities to freely organize and play, parents are able to give their children authentic opportunities to develop and practice critical social–emotional skills.

Overall, parents can minimize evolutionary mismatch by providing a variety of learning opportunities outside of school, such as a mix of structured and unstructured activities, unstructured play, and exploration. Such activities align with children’s evolutionary predisposition for hands-on, social, experiential learning (Bjorklund & Ellis, 2014). Additionally, parents can support social learning by encouraging interactions with peers of all ages and cooperative problem solving. This social learning reflects the informal, group-based learning environments in which children evolved (Steinberg, 2001). All of these listed ideas lead to a more evolutionarily informed parenting style and decreased instances of evolutionary mismatch in childhood.

## 8. How Teachers Can Evolutionize the Learning Process

Teachers arguably play the most central and immediate role in evolutionizing the public education system. Teachers are the stakeholders who most frequently and directly interact with students in an educational capacity, and they make the majority of instructional decisions. As a result, teachers have the potential to immediately and significantly adapt daily instruction to be more aligned with students’ evolved learning mechanisms and reduce the adverse impacts of evolutionary mismatch on learning.

For teachers, the toolkit of strategies relates to ways in which they can modify or focus their instructional practices to make classroom learning—even of secondary skills—more evolutionarily aligned. The strategies discussed below and in the toolkit include creating collaborative learning opportunities, focusing on hands-on work and movement, allowing for the student voice in learning and assessment, fostering real-world connections of student learning, and allowing ample opportunities for play. Strategies go beyond the ideas of effective or quality teaching and instead use theory from evolutionary educational psychology to create a full framework of effective pedagogical strategies for teachers. None of these strategies are intended to be used in isolation. However, by using some combination of these evolutionarily relevant elements, teachers can help decrease adverse impacts from evolutionary mismatch and improve modern education.

### 8.1. Collaboration and Mixed-Age Interactions

In the EEA, education was not an independent endeavor—children would have spent each day in mixed-age groups engaging in play and exploration (Gray, 2013). In contrast, modern schooling places significant focus on independent work and assessments in age-segregated classrooms. The hyper-focus on individualized work is to the detriment of our students and, when possible, should be replaced with opportunities for collaboration with peers of varying ages and abilities.

Collaboration or social learning involves children learning skills and behaviors from peers through face-to-face interactions (Samuelsson, 2023). To be most aligned with learning conditions in the EEA, groups should be small, with children of mixed ages. This type of learning functions as a sort of natural apprenticeship; younger children learn through observing, conversing, and working with older and more advanced peers. Younger children are able to learn and play within their zone of proximal development (in other words, what a child is able to do with support of a more knowledgeable other) by working with those older or more advanced peers on an activity they would not be able to achieve independently (Vygotsky, 1978). This collaboration is essentially a natural form of scaffolding (Gray, 2013). The process of working with those who are more advanced will lend itself to the modeling of the advanced behavior, which can then be replicated independently by the younger, less advanced child.

This collaboration is not only beneficial to the younger child, but to the older child as well, as it provides an opportunity to test what they know and therefore further their understanding of the material/task (Gray, 2011). Additionally, working in mixed-age groups allows for the older children to nurture and care for the younger children, thereby helping to develop kindness and empathy in the older children and provide a meaningful support system to the younger children (Gray, 2011).

Lastly, since there is a diminished sense of competition across ages—older children have nothing to gain from “beating” the younger children, and the younger children know they cannot “beat” the older children—age-mixing fosters more positive social interactions between students than those often seen in single-aged interactions (Gray, 2011). Younger children try to emulate desired activities and behavior of the older children, and the older children will have the opportunity to be more playful and creative. Age-mixing allows this to happen without judgment or competition. These interactions also have positive implications for lessening common school social issues, such as bullying (Volk et al., 2022).

It may not be feasible to engage in mixed-age opportunities often during the traditional school day without systemic change. So much of the education system is based on age-based standards. However, creating experiences when possible—such as mixed-age reading buddies, clubs, lunch periods, etc.—can help students experience some of the associated benefits of working with peers of different ages. Teachers can also create similar experiences more frequently by allowing students to work with their same-age peers within the classroom in heterogeneous ability groupings. This work could be performed while working on projects, during reading or math groups, or while playing classroom learning games. Overall, the more we are able to have children work together—either in mixed-age or mixed-ability groups—the more opportunities they have to learn meaningfully from other children and thus reduce the effects of the evolutionary mismatch of age-stratified classrooms.

### 8.2. Active, Hands-On Learning

Another crucial element of learning in the EEA was the emphasis on hands-on, active learning. Ancestral children likely learned all skills through practice and play with real-world tools (Gray, 2013). The doing aspect was critical to learning. Our current curricula often require children to sit and learn standardized material with very few opportunities for movement or hands-on exploration. In this form of passive learning, children today are expected to master skills solely through discussion and modeling from an adult (Petress, 2008). This passive experience is highly unnatural, compared to how they evolved to learn, as has been discussed at length so far in the present work. From an evolutionary lens, it is important that schools equip teachers with the necessary materials and knowledge to support active learning for students.

Students need access to materials that align with primary skills and help to develop essential abilities, such as social interaction and critical thinking. Physical play manipulatives, such as bean bags, puzzles, and building blocks, enhance motor, spatial awareness, and cognitive problem-solving skills (Møller, 2015). Fantasy play materials, such as dolls and board games, encourage children to role-play and mimic everyday life situations, thus promoting cooperative learning and conflict management (Hashmi et al., 2020). Social–emotional skills should be nourished through play and strategic games that teach empathy, negotiation, and conflict resolution—skills that were essential in our evolutionary past and remain so today. These tools and toys should be valued just as highly in elementary classrooms as academic tools.

Further, classrooms should also have access to materials designed to teach and enforce secondary academic skills through hands-on exploration. In modern elementary schools, these resources typically include items such as STEM kits (e.g., robotics and chemistry sets), mathematics tools (e.g., counting cubes and fraction models), and language learning tools (e.g., flashcards and letter tiles) (Cameron, 2020). Having access to tools to support the curriculum helps to foster a multisensory approach to learning, similar to that of the EEA, and thereby supports greater academic outcomes for students (Seidl et al., 2024; Broadbent et al., 2018; Schlesinger & Gray, 2017). Additionally, allowing students to explore concepts with hands-on tools prior to instruction increases motivation for children and allows them to take ownership over their academic learning (Bonawitz et al., 2011).

Altogether, both primary and secondary skill-building materials are crucial for supporting the development of the whole child, who can thrive in the modern classroom and succeed within the complexities of the world around them. Pen-and-paper workbooks, teacher lecturing, and drill and practice are unnatural to children’s evolved learning mechanisms and thereby ineffective on their own. Access to manipulatives and toys in the classroom can reduce evolutionary mismatch and help foster social–emotional skills, increased interest, and higher achievement in academic areas.

### 8.3. Student Voice

There were no adult teachers or required curricula for the vast majority of evolutionary history. Instead, children fully dictated their own learning based on their interest and the relevance of skills. Teachers today must follow dictated curricula or, at the very least, decide what to teach children and when to teach it. Children, as a result, have little say in their education. Results of this lack of input include disengagement with material and lower academic outcomes (Jones & Hall, 2022).

Conversely, alternative school models that engage in democratic methods have reported that students are more engaged and place a greater focus on their education (Gray, 2013). For example, in the Sudbury Valley School, which was previously discussed, students are fully involved in the democratic leadership of the school. Children, regardless of age, get a vote in all decision making, including allocation of resources and staffing decisions. Individual students also have the freedom to make decisions regarding what to learn, whether through activities, projects, or exploration. This work is all driven by innate curiosities and motivation. Teachers only step in when requested (Gray, 2017). In Montessori schools, the classroom environment is intentionally designed to encourage independence and agency, allowing children to choose their own activities and work at their own pace (Lillard, 2013). The self-directed approach closely mirrors how children learn under ancestral conditions (Gray, 2017).

In traditional public schools, choice is less frequent, as the mandated curriculum drives instruction. However, one place where choice can be given within reason is in project-based learning. When students are given agency to choose a topic, method of presentation, materials, etc., they have more ownership over their own learning and higher interest in the academic content (Fitzgerald, 2020; Tris & Andy, 2024). Projects are also a more evolutionarily aligned form of assessment than standardized testing, as projects are performed with less focus on regurgitation of information and more focus on practical use of skills (Friesen, 2010).

Inclusion of the student voice can also be supported by subject areas that are in addition to standardized curriculum. In elementary years, these subjects typically include music, art, and physical education. Library, technology, and robotics are also offered in many districts. While students benefit from being exposed to all the above areas, students often find their “niche” in one of these areas. By offering enrichment or club opportunities related to student interests, students are able to take ownership over a portion of their time in school, thereby making school a more enjoyable endeavor. The inclusion of the student voice addresses the mismatch of adult-directed curriculums.

### 8.4. Real-World Connections

Under ancestral conditions, all learning happened in the context of an individual’s small tribe. Children saw the immediate applications of what they were learning and were thereby motivated to practice and master different skills. In modern education systems, there is an implicit message that schools are institutions for learning, and thus, learning only happens within a school building. Schooling, albeit unintentionally, functions to decrease lifelong learning and educational engagement by downplaying the importance of the work and signaling that learning is for children only (Gray, 2013).

In modern classrooms, secondary skills take precedence over primary skills. The acquisition of secondary skills is likely more driven by the needs and motivations of society than the needs and motivations of children themselves (Geary, 1995). Matching skills to the real-world context can motivate the learning of more advanced skills. Take, for example, reading: Children need phonemic awareness, phonics, vocabulary, fluency, comprehension skills, and more, just to be effective readers (Scarborough, 2001). These skills are primarily secondary skills that require explicit instruction. However, children are generally motivated to learn to read. David Geary argues that reading and literacy are likely motivated by the content of what is being read rather than the process of reading itself. The content of books, especially children’s literature, often relates to primary knowledge and evolutionarily relevant themes such as social relationships (Geary, 1995). The same interest-based reasoning can be inferred to drive students’ interest in technology (Mitra, 2003). It is also likely that children are partially motivated by reading because they can clearly see the quantity of text and applied use of reading in their day-to-day lives, both in and out of school. This prevalence signals that reading is a necessary skill that, despite its difficulty, must be acquired (Neumann & Hood, 2009). In addition to reading, meaningful context has also been shown to positively impact mathematical understanding (Lepper & Henderlong, 2000). Simply, while our model evolutionizing the education model may often emphasize primary skills, we also suggest that primary and secondary learning are often intertwined.

By contextualizing learning of secondary skills for students, teachers are able to signal importance for academic learning. For example, understanding that multiplication can be used while shopping for groceries makes the repeated and tedious work of memorizing facts more meaningful for students. The same can be said for understanding that editing is something that publishers do, scientists research their topic before conducting experiments, etc. Contextualization in the real world helps decrease mismatch, motivate students to engage in academic or other abstract tasks, and inspires children to develop as life-long learners.

### 8.5. Play

Learning for ancestral children took place nearly exclusively through play (Gray, 2009). While the skills that modern children must learn to be successful are more numerous and complex than those of ancestral children, childhood remains a time that is characterized by a high interest in play and exploration. Current research shows that play remains a primary driver of childhood learning (Pellegrini, 2009).

During play, both physical and cognitive processes are employed. Through play, children can test social skills and cultural roles, develop executive functioning skills, and improve fine and gross motor skills (Samuelsson, 2023). All of this happens in an environment that is less risky than real-world experience. There are various types of play that can be leveraged in the classroom to support academic and/or social emotional learning. For the sake of this paper, free play or pretend play, explorative play, and guided play are highlighted.

The first type of play, free play or pretend play, allows children to decide what they are interested in investigating by partaking in “as if” scenarios that pique the child’s internal interest. Thompson and Goldstein (2019) explained the importance of pretend play by pointing out that, in order to properly engage in it, children not only need to be able to share the experience of their pretend world, but also need the ability to communicate in advanced ways to other players. This play requires that children naturally practice communication, flexible thinking, role-taking, and executive functioning (Lancy & Grove, 2011). Children will not learn all required curricular skills through free play, but they will practice necessary social–emotional skills that will help support readiness for academic learning. This type of play would primarily be seen through time outdoors on the playground or while playing with dolls, kitchen supplies, etc. in the classroom.

Another type of play, exploratory play, sets the stage for academic learning. By engaging with items in a hands-on manner, students create concrete understandings of abstract concepts. It has been shown that, when children explore prior to instruction, or in the absence of instruction, they are more likely to express greater and more prolonged interest. They are also more driven to come to their own conclusions and discoveries (Bonawitz et al., 2011). By providing play and inquiry-based lessons, teachers can increase engagement with academic concepts for their students. This type of play could be as simple as letting children explore math manipulatives prior to using them for a lesson or putting out literacy tools, such as letter tiles, for exploration.

Lastly, guided play can be used in modern classrooms. Guided play includes games or explicit teaching during free play episodes (see Bjorklund, 2022). While this type of play is mostly restrictive and directed, it still capitalizes on children’s evolved playfulness and allows for academic work to be approached in a motivating and safe manner. Academic games can allow for the practice of academic skills, as well as supporting social–emotional development (Petersson & Weldemariam, 2022; Berson et al., 2023). By tapping into children’s innate desire for play, teachers can decrease evolutionary mismatch and improve learning in their classrooms.

Overall, by incorporating evolutionary educational psychology techniques, such as mixed-age or collaborative groups, active learning, student choice, context for learning, and play into modern day classrooms, teachers would lessen adverse impacts from evolutionary mismatch. As a result, they would likely see improvements in not only how well students perform academically, but also in how they behave and their overall enjoyment of school.

## 9. How Administrators (At Different Levels) Can Evolutionize the Learning Process

As educational institutions wrestle with how to navigate the diverse needs of modern learners, it becomes evident that conventional methods often do not align with the innate ways in which humans evolved to learn (Gruskin & Geher, 2018). This misalignment highlights the necessity for educational administrators to rethink their approaches by incorporating evolutionary principles into their frameworks. Educational administration refers to the organization and management of academic institutions to ensure effective teaching and learning environments (Trinidad, 2024). The responsibility of administrators is to oversee areas including policy development, budgeting, staffing/hiring, and adherence to state/federal regulations.

It is important to acknowledge that multiple levels of administration are involved in these processes, each responsible for different aspects of decision-making. Considering education through an evolutionary lens would benefit administrators by helping to create more evolutionarily matched learning facilities and practices that not only address the diverse academic needs of students but also promote social learning and resilience (Bjorklund, 2021). Embracing evolutionary principles allows for the development of adaptive strategies that foster collaboration among educators, encourage flexible curricula tailored to individual interests, and use empirical evidence to improve instructional effectiveness.

### 9.1. Federal and State Level Administration Strategies

According to *evolutionary leadership theory*, there is a discrepancy between modern and ancestral organizational environments, due to evolutionary mismatch (van Vugt & Ronay, 2013). Evolutionary mismatch in learning conditions has led to increased challenges in the world of education and academia. Therefore, establishing an adaptive framework is essential for educational administrators who seek to implement evolutionary principles in their institutions (Geary, 2008). This work begins with setting a clear vision and policies at both the federal and state levels that align with the evolved needs of students.

Although American society often looks to the federal government to take charge, it is actually state governments that determine most educational policy (Bowman, 2018). Federal laws concerning education pertain to equal access and safeguarding students’ and teachers’ constitutional rights. It is important that policy be made that keeps in mind the need for teachers to have freedom to create evolutionary friendly lesson plans and instructional decisions that are conducive to student development and needs. At the federal level, the government also supports elementary education by providing funding through programs such as “Title I” that help schools that have a high percentage of low-income students. Allowing local administrators the flexibility to use resources in an adaptive manner that addresses the needs of different student populations ensures that schools can adapt to their students’ needs and improve to better serve varying communities. In short, the federal government helps support the autonomy of more local administrators to decrease levels of evolutionary mismatch in their schools and provide a more evolutionarily aligned educational experience.

States hold a great deal of power over education (Bowman, 2018). States are responsible for key tasks, such as overseeing the maintenance and operation of public schools, establishing curriculum requirements and grade-level standards, regulating teaching methods, and overseeing additional aspects of the education system. Each state has its own standards and policies that directly impact the quality of education available to students (Moore et al., 2003). However, when educational policies and standards differ significantly from state to state, it is difficult to ensure a high-quality and equitable education for all students, regardless of their location. These differences can pose additional challenges for students who transfer schools or participate in dual programs, thereby exacerbating challenges from evolutionary mismatch. It would, therefore, be beneficial for educational administrators to integrate evolutionary theory at both the federal and state level, thereby ensuring that all schools are united in utilizing evolutionary principles in their frameworks and policies. Potential policies could include some degree of overseeing learning standards, safeguarding time for recess, replacing standardized testing with more evolutionarily aligned means of assessment (i.e., performance tasks or portfolios), and an emphasis on social–emotional learning standards. These potential policies work to reduce evolutionary mismatch and thereby create better outcomes for modern students.

### 9.2. District-Level Administration Strategies

In the context of district-level administrators (e.g., Board of Education members and superintendents), resource allocation plays a crucial role in ensuring equitable access to materials and technology, so that all students can benefit from an evolutionarily relevant classroom environment. Evolutionarily relevant classrooms reduce evolutionary mismatch by mimicking key aspects of the EEA with natural materials, flexible furniture, and spaces that support collaborative interactions and access to outdoor exploration. Funds can also be used to purchase learning materials that are developmentally appropriate, multisensory, and allow for more evolutionarily relevant learning experiences (e.g., puzzles, letter tiles, blocks, and math manipulatives).

Administrators can seek grants and partnerships with local businesses or educational organizations to supplement district funding. Administrators can also involve teachers in budget discussions to identify specific needs and ensure that funding is directed toward goals that will have the greatest impact on teaching and learning. Funding can support student growth at its current levels by providing targeted interventions tailored to each student’s needs, in line with Vygotsky’s Zone of Proximal Development (ZPD) (Shabani et al., 2010). In particular, funding can be used to hire specialists, such as special education teachers, therapists (e.g., occupational therapists or physical therapists), and classroom aides who can work closely with students and provide individualized support.

Funding for professional development is equally important, as it enables educators to develop and refine their teaching methods over time (Darling-Hammond & McLaughlin, 2011). Administrators can prioritize budget allocations for professional development that focuses on teacher training, class resources, and technology through an evolutionary lens. Such training would allow teachers to better understand the ideas of evolutionary educational psychology. As outlined above, such understanding would allow teachers to understand and decrease evolutionary mismatch in the classroom, thereby leading to better outcomes for modern students.

### 9.3. School-Level Administration Strategies

At the school level, administrators (e.g., principals) play a crucial role in fostering a culture of collaboration—which is essential for enhancing educational outcomes. From the perspective of evolutionary psychology, the school setting can be viewed as a local tribe or community of alloparents, collectively caring for the needs of children (Emmott & Page, 2019). Alloparenting refers to any individual who plays a caregiving role to their non-biological offspring—e.g., friends, babysitters, and educators (Hrdy, 2009). Encouraging teamwork among teachers strengthens instructional practices, but also taps into the natural human propensity to gain insights and advance through social interactions. From an evolutionary perspective, working in groups—such as a school community—highlights the intrinsic tendency to work together, which has been essential for survival throughout human history (Van Vugt & Schaller, 2008).

In contemporary settings, such as educational institutions, group work promotes the exchange of complex ideas that lead to more effective problem-solving and strategies. Creating opportunities for educators to engage in peer-learning groups through regular team meetings, focus groups, and peer observations, allows educators to share best practices and develop collectively, thereby forming a supportive network that mirrors evolutionary principles of cooperative behavior (Miquel & Duran, 2017). Additionally, implementing flexible and diverse models of education—such as project-based and holistic learning—allows for more personalized lessons, catering to student’s innate curiosity and interests, much like how humans have historically learned through exploration. By adapting elements from evolutionarily aligned education systems—such as Sudbury or Montessori—administrators can adapt methods at their own schools to meet the diverse needs of children and give them responsibility and control over their learning (Lillard, 2013; Gray, 2013).

By embracing flexibility in scheduling, lesson delivery, curriculum preparation, and classroom design, administrators have the opportunity to revolutionize educational models. The current rigid adherence to a predetermined curriculum with a specific timeline for instruction not only stifles creativity but is also inconsistent with the principles of evolution that are essential for modern education. Moving towards an evolutionary-friendly approach, from all levels of administration, will help decrease evolutionary mismatch and improve educational outcomes for all students.

## 10. Discussion

There are few human endeavors as important as the education of our children. The obligatory public school model, focusing on secondary knowledge (Geary, 2008), emerged within the past few centuries to match the changing landscape of the industrialized world. This shift, which includes a large focus on secondary knowledge, mismatches the kinds of learning environments that surrounded the deep evolutionary history of our species. Based on this large-scale evolutionary mismatch (see Gruskin & Geher, 2018), this approach to education neglects various important features of human development (such as social and emotional development) in favor of aspects of human development that only emerged more recently in human evolutionary history. As described herein, mismatches of various kinds abound, including the fact that children are mostly taught by adults, primary knowledge is downplayed, group work is downplayed, classrooms are segregated by age, and children lack time for play. People who report having had educational experiences that better match modern nomadic (and, likely, ancestral human) conditions tend to report more favorable experiences and they tend to perform more favorably in modern academic contexts, relative to their more mismatched peers

This paper provides guidance to three critical stakeholders in helping to bring evolutionarily relevant features to modern educational contexts, in an effort to help students in the industrialized world reap benefits of factors that surrounded the kind of education that our ancestors evolved to experience. By addressing the evolutionary mismatch in modern education, stakeholders can create systems that better align with evolved cognitive and social developmental mechanisms.

Parents would benefit from learning the basic idea of evolutionary mismatch, along with basic mismatches that exist in modern, industrialized-world schooling (such as understanding why things such as recess, art, and music should get more front-and-center attention in their school districts). It is also key for parents to develop an evolutionarily informed parenting style to support the work that is done in schools. Teachers can benefit from using the toolkit provided for them to help them not only understand the connections between mismatch and education, but to understand specific strategies and approaches (such as focusing more on group work and projects and less on pen-and-paper work, connecting classroom learning to real-life contexts, and creating ample opportunities for play in school) to help them shape classrooms that better match the ways that children evolved to learn and develop. Finally, school administrators can certainly benefit from understanding mismatch and its many applications to education. Further, these particular stakeholders have the power to make policy-based changes that can institutionalize evolutionarily relevant elements of education systemically, ensuring that students across all educational levels have the opportunities to benefit from the kinds of teaching and learning practices that surrounded the evolutionary history of humans.

There remains work to be done and questions that must be answered regarding addressing evolutionary mismatches and creating more evolutionarily informed schools. Further research should be conducted to better understand the broader implications of any changes to the educational system. As many stakeholders are resistant to change within the system, better education regarding evolutionary educational psychology is necessary for all stakeholders. The question then becomes how to best provide this type of critical education. Lastly, it is important to consider potential barriers to these ideas and how stakeholders can be expected to overcome these barriers.

Overall, the evolutionary perspective on the human condition provides exceptional and novel insights into so many spheres of life. In recent years, educational processes have been examined extensively from an evolutionary perspective (see Gray, 2011). Taking our evolved history into account when shaping educational practices can (as demonstrated by Gruskin & Geher, 2018) have benefits that are not only related to students’ enjoyment of the school experience, but may also lead to relatively positive academic outcomes. The evolutionary approach to education has the potential to be highly beneficial for students and can be feasibly implemented within the constraints of the modern school system. The toolkit provided herein provides realistic guidance on implementing critical evolutionarily informed changes. We urge all stakeholders in the educational process to take the positive outcomes outlined in the current work under serious consideration, for the benefit of students and educators today and for generations to come.

## Data Availability

Not Applicable.

## Appendix A

Stakeholder Toolkit: Use the following strategies and prompts to reflect on your role in evolutionizing education. Ensure you describe how you would address the question prompt. Adding potential examples would be helpful.

Parents:		
Become educated in the education/evolution interface.		
Upbringing and Home Support	→ Encourage technological boundaries at home (e.g., limiting screen time and replace screen-based activities with hands-on activities). → Stay involved with your child’s holistic school experience (academics, play, and social-emotional learning).	Prompt: What features of your home life can you change to make things more evolutionarily natural for your child?
Childhood Empowerment	→ Support the development agency by allowing your child to make choices related to required academic (e.g., homework scheduling) and nonacademic activities (e.g., creative hobbies) within practical parameters. → Provide options in involving children in important life skills such as cooking and cleaning to develop general self-efficacy.	Prompt: How can you help provide choices for your child’s academic and non-academic activities that take place at home?
Unstructured Activities	→ Allow your child to explore their interests, physical environments, and social environments. → Provide reasonable parameters regarding unstructured play for safety related purposes.	Prompt: What are some changes that you can make in parenting your child that safely increase the amount of unstructured play they experience?

Teachers:		
Become educated in the education/evolution interface.		
Collaboration	→ Create opportunities for mixed-age reading buddies. → Allow for work and assessments based on mixed ability projects.	Prompt: How can you convert an individualized assignment into a collaborative experience?
Active, Hands-On Learning	→ Design lessons and work using hands-on manipulatives and tools. → Create movement opportunities during lessons and work periods.	Prompt: How can you take a traditional lesson and convert it into an active learning experience?
Student Voice	→ Create project project-based assignments rather than worksheets or tests. → Allow students to choose their topics when appropriate (e.g., passion projects).	Prompt: How can you convert teacher-directed work into student-directed work?
Real-World Connections	→ Connect abstract ideas and concepts with their practical uses (e.g., multiplication helps with grocery shopping). → Create opportunities for students to practice skills in context through role-play or free play.	Prompt: How do you convert an abstract topic into a skill with a real-world application?
Play	→ Encourage free play, exploratory play, and guided play. → Make time for outdoor play activities (e.g., recess).	Prompt: How can you budget time to ensure time for both academics and play are provided for students?

Administrators:		
Become educated in the education/evolution interface.		
Government Level Administrators	→ Budget federal or state funds to support the work done by teachers to evolutionize classrooms. → Use evolutionarily informed decision-making when developing educational policy.	Prompt: What current policies work against students’ evolved learning inclinations, and how can change be made to better align these policies with evolved learning inclinations?
District Level Administrators	→ Budget district funds for classroom furniture and tools that supports evolutionarily natural learning environments. → Development district level policies and professional development that foster evolutionarily relevant instruction.	Prompt: What current policies work against students’ evolved learning inclinations, and how can change be made to better align these policies with evolved learning inclinations?
School Level Administrators	→ Encourage teachers to collaborate to develop evolutionarily informed practices. → Allow teachers flexibility in scheduling and instructional methods to implement evolutionarily informed practices.	Prompt: What current policies work against students’ evolved learning inclinations, and how can change be made to better align these policies with evolved learning inclinations?
